# High-Resolution Imaging by Adaptive Optics Scanning Laser Ophthalmoscopy Reveals Two Morphologically Distinct Types of Retinal Hard Exudates

**DOI:** 10.1038/srep33574

**Published:** 2016-09-19

**Authors:** Muneo Yamaguchi, Shintaro Nakao, Yoshihiro Kaizu, Yoshiyuki Kobayashi, Takahito Nakama, Mitsuru Arima, Shigeo Yoshida, Yuji Oshima, Atsunobu Takeda, Yasuhiro Ikeda, Shizuo Mukai, Tatsuro Ishibashi, Koh-hei Sonoda

**Affiliations:** 1 Department of Ophthalmology, Graduate School of Medical Sciences, Kyushu University, Fukuoka, Japan; 2 Retina Service, Department of Ophthalmology, Massachusetts Eye and Ear Infirmary, Harvard Medical School, Boston, Massachusetts, USA

## Abstract

Histological studies from autopsy specimens have characterized hard exudates as a composition of lipid-laden macrophages or noncellular materials including lipid and proteinaceous substances (hyaline substances). However, the characteristics of hard exudates in living patients have not been examined due to insufficient resolution of existing equipment. In this study, we used adaptive optics scanning laser ophthalmoscopy (AO-SLO) to examine the characteristics of hard exudates in patients with retinal vascular diseases. High resolution imaging using AO-SLO enables morphological classification of retinal hard exudates into two types, which could not be distinguished either on fundus examination or by spectral domain optical coherence tomography (SD-OCT). One, termed a round type, consisted of an accumulation of spherical particles (average diameter of particles: 26.9 ± 4.4 μm). The other, termed an irregular type, comprised an irregularly shaped hyper-reflective deposition. The retinal thickness in regions with round hard exudates was significantly greater than the thickness in regions with irregular hard exudates (P = 0.01 →0.02). This differentiation of retinal hard exudates in patients by AO-SLO may help in understanding the pathogenesis and clinical prognosis of retinal vascular diseases.

Retinal vascular diseases including diabetic retinopathy (DR) and retinal vein occlusion (RVO) are one of the most common causes of blindness among adults of working age in developed and developing countries^1^,^2^. Retinal findings in these vascular diseases include microaneurysms, hemorrhages, and hard exudates. Hard exudates are bright, reflective, white or yellow lesions in the retina that are indicative of increased retinal vascular permeability and edema^3^. The components of hard exudates from autopsy specimens have been histologically examined by several groups. In 1957, Wolter *et al*. reported an accumulation of fat-filled phagocytes, and hyalinization of lipids was observed in retinas with hard exudates from two patients with diabetic and angiospastic retinopathy^4^. In addition, Toussaint *et al*. observed hyaline material in retinas from patients with DR and PAS-laden macrophages that were often stained with lipid-detecting Sudan staining^5^. A recent immunohistochemical study by Cusick showed that CD68(+) macrophages were detectable by oil red O staining in hard exudates in DR^6^. These histological studies indicate that hard exudates include macrophages or noncellular material such as lipids or proteinaceous substances (hyaline substances). However, there have been no studies correlating features of these hard exudates with clinical characteristics.

Retinal imaging techniques have undergone rapid and significant improvements in recent years. Adaptive optics scanning laser ophthalmoscopy (AO-SLO) is a new technique that enables observation of the retina at the cellular level in patients^7^. Although AO-SLO was designed to visualize photoreceptors and has been mostly employed for this purpose, it has also been applied to the visualization of vascular features in both normal retina and retina in vascular disorders^8^,^9^. Therefore, AO-SLO has been used to investigate various retinal diseases at high magnification and has led to new knowledge in retinal disorders^10^,^11^,^12^,^13^. A few studies using AO imaging have observed the microscopic structure of hard exudates in the retina of living subjects^10^,^14^,^15^. However, these studies have not investigated relationships between the AO image and clinical characteristics or previous pathological observations.

We hypothesized that the morphological characteristics of hard exudates observed in pathology specimens were related to the activity of the retinal vascular disease. Therefore, in this study, we investigated the morphological characteristics of hard exudates in patients with retinal vascular diseases using AO-SLO, in an attempt to correlate these findings with clinical characteristics and histological data previously described in the literature.

## Results

**Figure 1:**
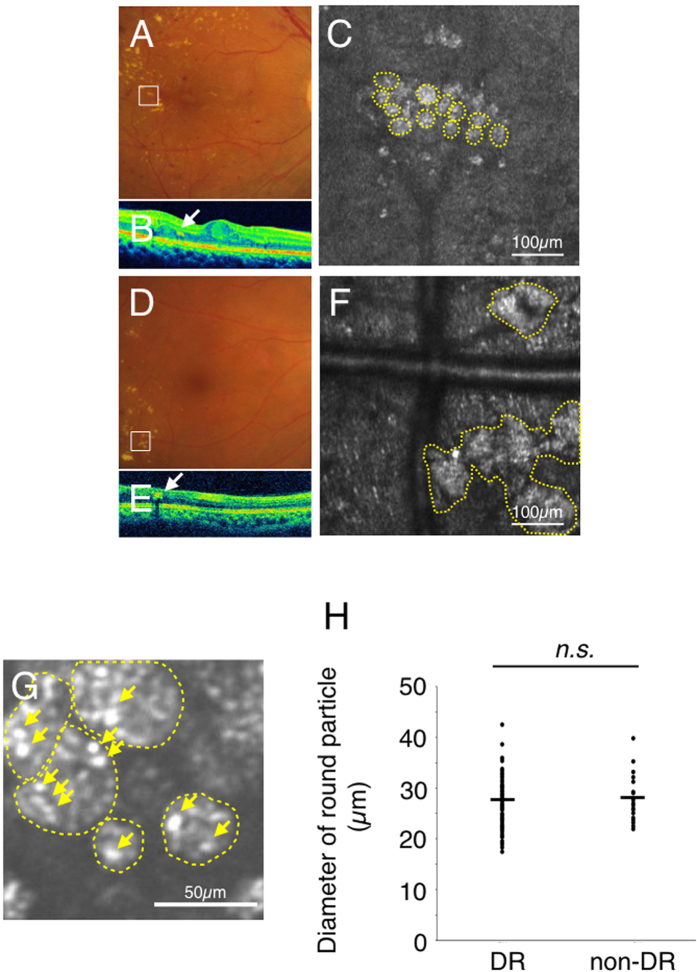
Two types of hard exudates in an adaptive optics scanning laser ophthalmoscopy (AO-SLO) image. Case 1 with diabetic retinopathy (**A–C**), case 20 with diabetic retinopathy (**D–F**), and case 16 with diabetic retinopathy (G). (**A,D**) indicate the color fundus photograph (white squares: imaged area of AO-SLO). (**B,E**) indicate spectral domain optical coherence tomography (SD-OCT) image (white arrows: hard exudate). AO-SLO image shows accumulation of spherical particles (**C**) and irregular shaped foci (**F**). Yellow dotted circles indicate each spherical particle (**C**) and irregular hyper-reflective deposition (**F**). G shows detailed observation of spherical particles (yellow dotted circles) in AO-SLO image. The diameter of these spherical particles was 26.9 ± 4.4 μm (*n* = 140). Yellow arrows indicate some hyper-reflective dots in spherical particles (**G**). Comparison of the diameter of spherical particles in hard exudates in diabetic retinopathy and non-diabetic retinopathy. There is no significant difference between diabetic retinopathy (DR) and non-diabetic retinopathy (non-DR) (**H**). *n.s.*: not significant, the Wilcoxon matched-pairs signed-ranks tests.

Patient characteristics in this study are summarized in Table 1. AO-SLO observation showed hard exudates as hyper-reflective patterns, and two distinct morphologies of hard exudates could be seen even though they could not be distinguished by color fundus photograph or by SD-OCT (Fig. 1A,B,D,E). One morphology consisted of an assembly of spherical particles each having a heterogeneous internal intensity (round type; Fig. 1C). The other hard exudate morphology comprised an irregularly shaped deposition with a hyper-reflective intensity (irregular type; Fig. 1F). The average diameter of spherical particles was 26.9 ± 4.4 μm (*n* = 140, Fig. 1G). Many small hyper-reflective dots with a wide range of reflective intensities could be observed in each spherical particle (Fig. 1G). There was no difference in the average diameter of these spherical particles between DR and non-DR retinal diseases (Fig. 1H).

**Figure 2:**
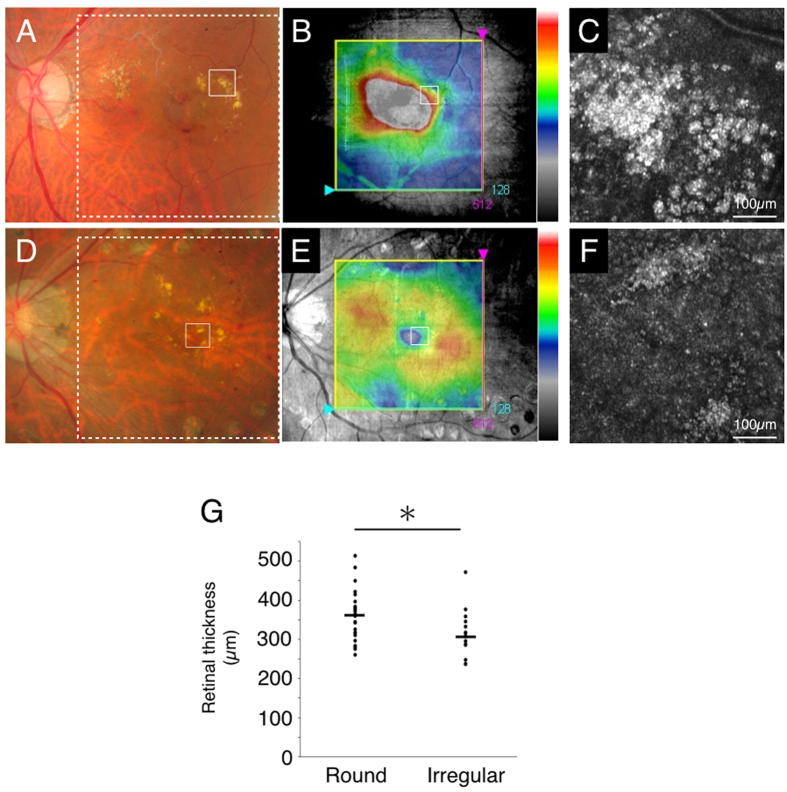
Retinal thickness in regions associated with the two types of hard exudates. Case 27 with branch retinal vein occlusion (round type, **A–C**) and case 3 with diabetic retinopathy (irregular type, **D–F**). (**A,D**) indicate the color fundus photograph (white solid squares: imaged region of adaptive optics scanning laser ophthalmoscopy (AO-SLO), white dotted squares: imaged region of spectral domain optical coherence tomography (SD-OCT) in B and E, respectively). (**B,E**) indicate SD-OCT image with the macular cube protocol (128 horizontal line raster with 512 A-scan each, within a 6 × 6 mm^2^ area). (**B,E**) Retinal thickness (from ILM to RPE) was displayed geographically as a false-color topographic map. White solid squares in B and E indicate imaged region of AO-SLO in C and F, respectively. AO-SLO image shows accumulation of spherical particles (**C**) and irregular shaped foci (**F**). (**G**) Retinal thickness in the region associated with round type of hard exudates is significantly thicker than that with irregular type of hard exudates (*n* = 42, *P* = 0.02, the Wilcoxon matched-pairs signed-ranks tests).

To investigate if the morphology of hard exudates was associated with macular edema, the retinal thickness was measured in the region with hard exudates in SD-OCT. The correlation between the retinal thickness and the morphological type was analyzed. The retinal thickness in the region with the round type of hard exudates was significantly greater than that with the irregular type of hard exudates (*P* = 0.02) (Fig. 2).

**Figure 3:**
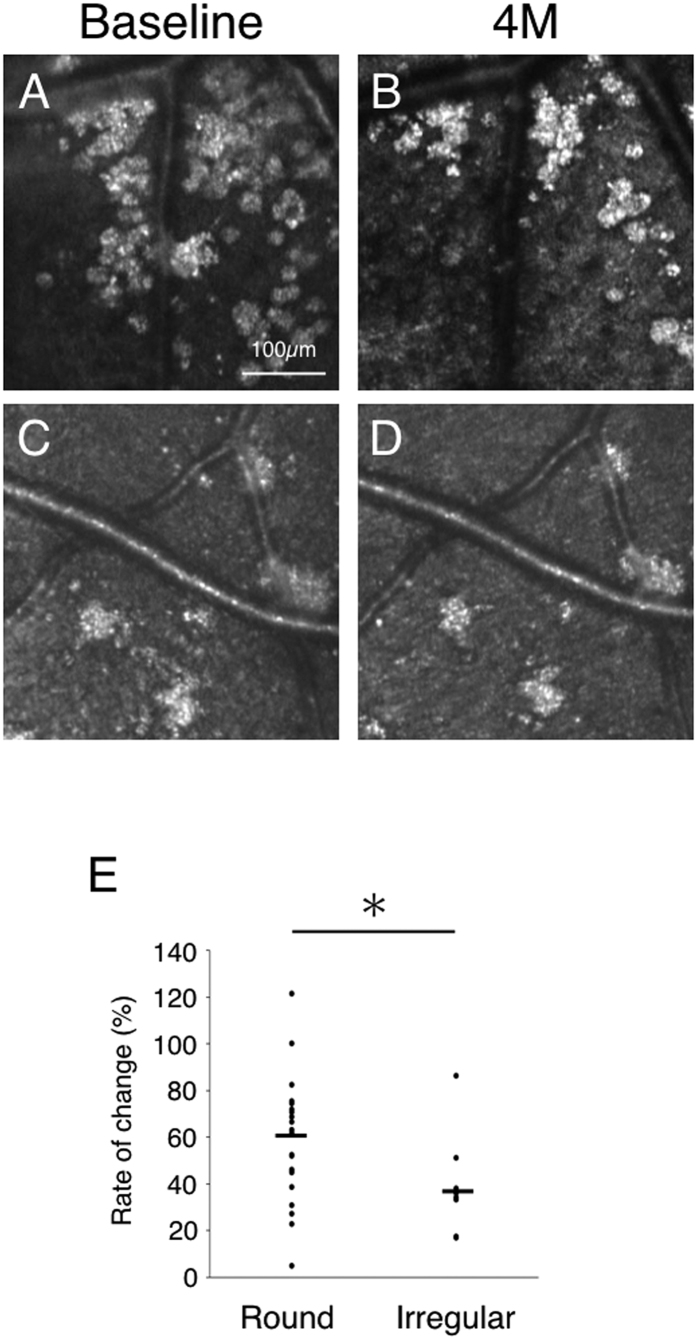
The follow-up of hard exudates with adaptive optics scanning laser ophthalmoscopy (AO-SLO). Case 10 with diabetic retinopathy at the first examination (round type, **A**) and four months later (round type, **B**). Case 28 with hypertensive retinopathy at the first examination (irregular type, **C**) and four months later (irregular type, **D**). The rate of change (%) was calculated from the change in area per hard exudate area during four months of follow-up relative to the first visit. (**E**) Comparison of the rate of change of the two types of hard exudates. The rate of change in round hard exudates is significantly greater than that of irregular hard exudates (*n* = 30, *P* = 0.02, the Wilcoxon matched-pairs signed-ranks tests).

It is known that some hard exudates eventually disappear while some hard exudates persist. To investigate if the morphology of hard exudates could be associated with its eventual disappearance, we reviewed the morphological type at the first clinical visit and at the subsequent visits. Thirty regions of hard exudates (21 round types and nine irregular types) were followed for four months and the rate of change in these regions was analyzed. The rate of change of the round type was greater than that of the irregular type (*P* = 0.02) (Fig. 3). This data suggests that the turnover of the round type is higher than that of the irregular type.

**Figure 4:**
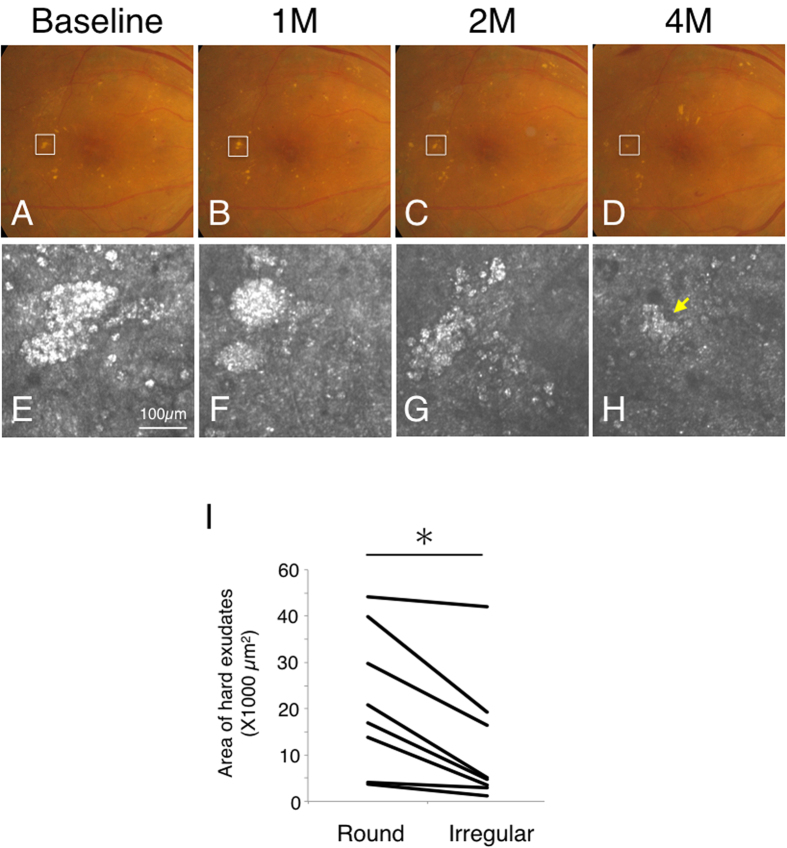
Changes in the two types of hard exudates. (**A–H**) Transformation of hard exudates (from round type to irregular type). Case 3 with diabetic retinopathy at the first examination (**A,E**), one month later (**B,F**), two months later (**C,G**) and four months later (**D,H**). White squares in color fundus photograph indicate imaged regions in adaptive optics scanning laser ophthalmoscopy (AO-SLO) (**A–D**). Hard exudates were observed as the round type by AO-SLO (**E**) at the first examination. After four months, the round type of hard exudates had transformed to the irregular type (yellow arrow) (**H**). (**I**) When the round type of hard exudate transformed into the irregular type, the area of hard exudates also decreased (*n* = 8, *P* = 0.006, Student t-test).

During follow-ups with AO-SLO, it was found that one type of hard exudate could change into the other type over time. To examine the pattern of this transformation between the two hard exudate types, we reviewed the type of hard exudate observed at the first visit and at the visit after four months (Fig. 4). Round type hard exudates were found to change into irregular type hard exudates (38%). For examples in case 2 with DR, round type hard exudates, which consisted of an assembly of spherical particles, were detected by AO-SLO at the first examination. However, four months later, these round type hard exudates had transformed into the irregular type (Fig. 4A–H). When these round type hard exudates transformed into the irregular type, the area of hard exudates also decreased (*P* = 0.006) (Fig. 4I). In case 2, the hard exudates disappeared six months after the first examination.

### Case Presentation

#### CASE 1

**Figure 5:**
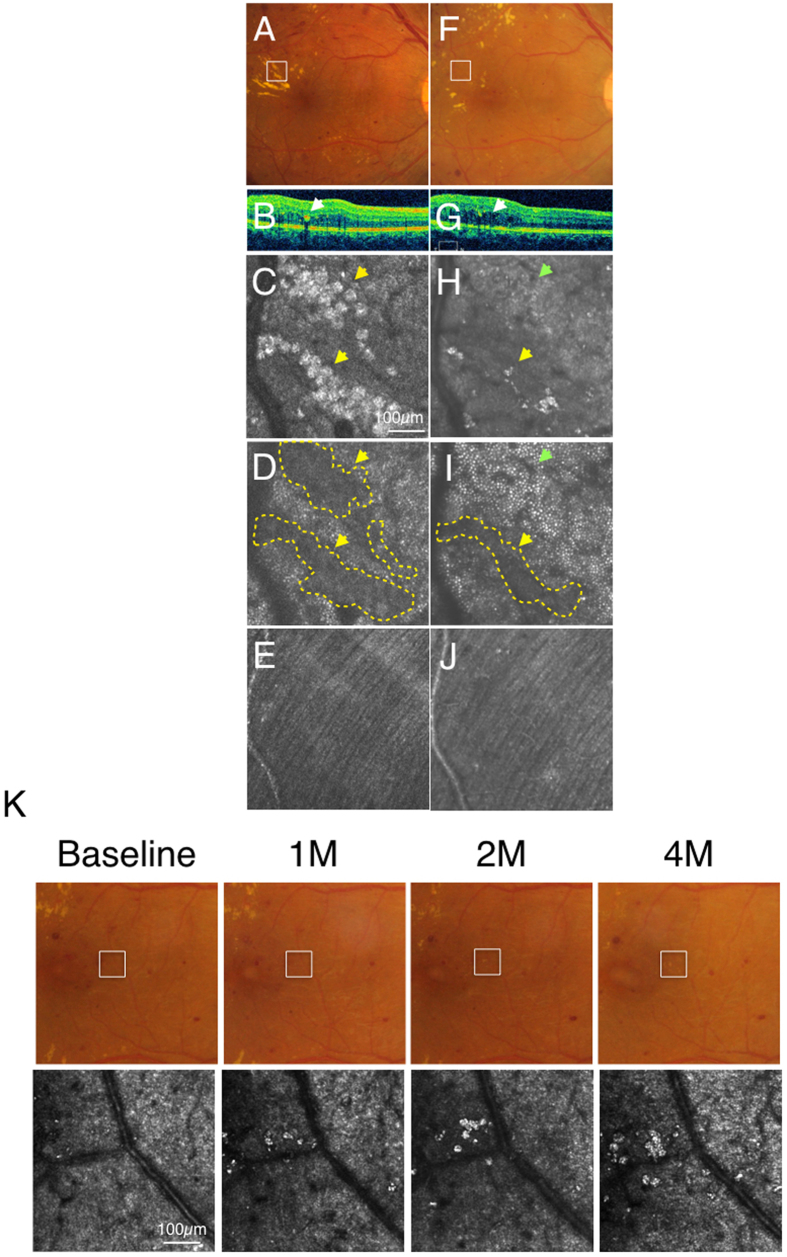
Case 1 (diabetic retinopathy): Color fundus photograph (**A**) and spectral domain optical coherence tomography (SD-OCT) (**B**) showed hard exudates in the outer plexiform layer and the outer nuclear layer (arrows). Adaptive optics scanning laser ophthalmoscopy (AO-SLO) focused and imaged layer with hard exudates (**C**), photoreceptor layer (**D**) and nerve fiber layer (**E**). When focusing at the layer with hard exudates, hard exudates could be observed as hyper-reflective objects (yellow arrows) at the intermediate layer (**C**). When focused at the photoreceptor layer, dark regions (yellow arrows, yellow dotted areas) could be seen corresponding to the shadow of hard exudates (**D**). Nerve fiber layer was not affected by hard exudates (**E**). After one year and four months, color fundus photograph (**F**), SD-OCT (**G**) and AO-SLO (**H**) showed withdrawal of hard exudates. Some dark spots could be still observed at the photoreceptor level (yellow arrow, yellow dotted area) and a cone mosaic could be observed in an area where a dark spot was observed at the first examination (green arrow) (**I**). Nerve fiber layer is still intact (**J**). (**K**) Formation of hard exudates in different regions. AO-SLO image and color fundus photograph with diabetic retinopathy at the first examination, one month later, two months later and four months later. White squares in color fundus photograph indicate imaged regions in AO-SLO. The number of spherical particles increased gradually and accumulated over time. In the color photographs, the spherical particles could be detected as hard exudates.

A 41-year-old man (HbA1c: 7.4%, duration of diabetic mellitus: three years) had nonproliferative DR in the right eye. The color fundus photograph of the right eye showed typical parafoveal hard exudates, which were located in the outer plexiform layer on SD-OCT imaging (Fig. 5A,B). On AO-SLO, hard exudates were observed as the round type (Fig. 5C). When focused on the photoreceptor layer, dark spots were observed as shadows corresponding to the region of hard exudates in the inner layer (Fig. 5D). No abnormal lesion was observed in the nerve fiber layer (Fig. 5E). In one area, the majority of hard exudates disappeared one year after the first examination (Fig. 5F,G). On AO-SLO, this corresponded to some dark spots at the photoreceptor layer returning to normal, with some that remained (Fig. 5H,I). The nerve fiber layer remained normal throughout during the follow-up with AO-SLO (Fig. 5J). In another area of the retina, the number of spherical particles increased gradually and accumulated over time. In color photography, it became detectable as hard exudates in the scan area as well (Fig. 5K).

#### CASE 24

**Figure 6:**
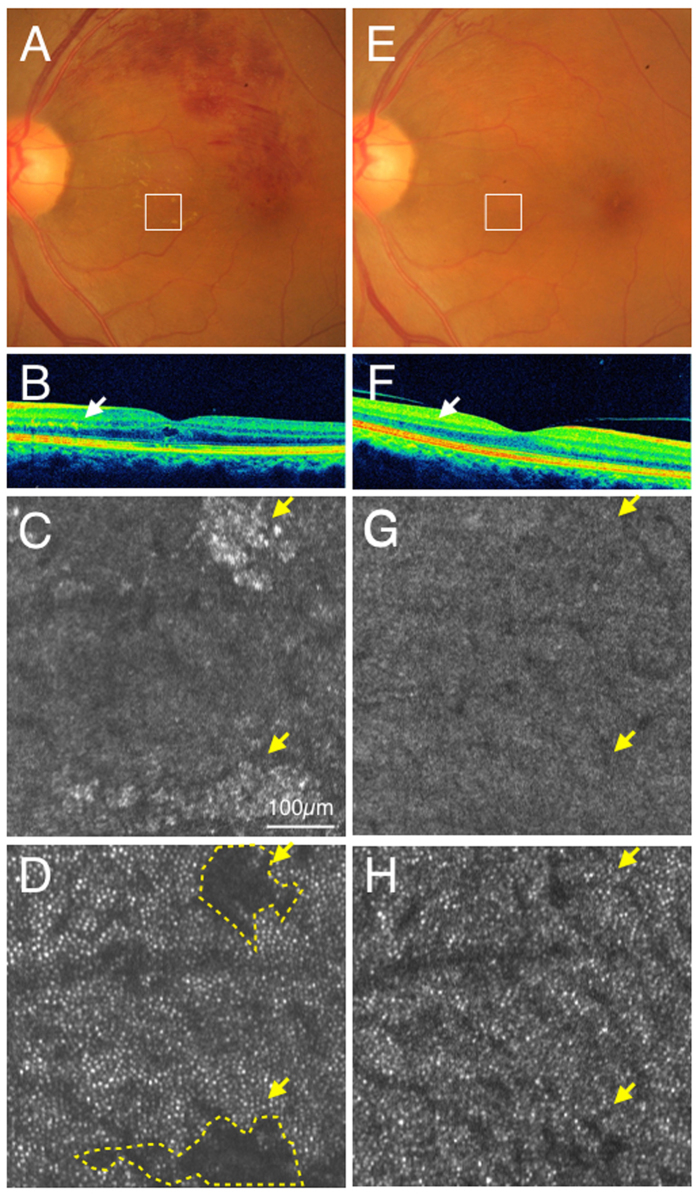
Case 24 (branch retinal vein occlusion): color fundus photograph (**A**) and SD-OCT (**B**) showed hard exudates in the outer plexiform layer (Arrows). Adaptive optics scanning laser ophthalmoscopy (AO-SLO) focused and imaged layer with hard exudates (**C**) and photoreceptor layer (**D**). When focused at the layer with hard exudates, hard exudates could be observed as hyper-reflective objects (yellow arrows) (**C**). When focused at the photoreceptor layer, dark areas (yellow arrows, yellow dotted areas) could be observed corresponding to the shadow of hard exudates (**D**). After one year and one month, color fundus photograph (**E**), spectral domain optical coherence tomography (SD-OCT) (F) and AO-SLO (**G**) showed withdrawal of hard exudates. A cone mosaic could be observed in an area where a dark spot was observed at the first examination (**H**). Yellow arrows indicate areas where hard exudates could be observed at the first examinations (**G,H**).

A 55-year-old woman had branch retinal vein occlusion (BRVO) in the left eye. In her case, dark spots could be observed in the photoreceptor layer that corresponded to the region of hard exudates in the inner layer (Fig. 6). Hard exudates (irregular type) disappeared during the thirteen-month follow-up, and AO-SLO image showed a normal cone mosaic in the photoreceptor layer (Fig. 6C,D,G,H).

## Discussion

Hard exudates are indicative of present or past retinal edema caused by leakage from the blood vessels due to breakdown of the blood-retinal barrier^3^. However, hard exudates have not been well characterized in patients due to the insufficient resolution of existing fundus examination and imaging techniques^16^. AO-SLO enables high resolution examination of the retina in patients with confocal imaging^7^,^8^,^9^,^10^,^11^. A few groups have observed hard exudates as a heterogeneous reflection or a hyper-reflective lesion with AO imaging^10^,^14^,^15^. In this study, we found that upon AO-SLO imaging, hard exudates could be divided into two morphologically distinct types with one having a spherical particle (round type) and the other having an irregular shape (irregular type). These round and irregular types of hard exudates distinguishable by AO-SLO imaging could not be differentiated by fundus examination, color fundus photograph, or by commercial SD-OCT imaging. This higher resolution of AO-SLO imaging enabled the analysis of hard exudates at the cellular level. Histological findings in the 1950’s–1960’s have suggested that lipid-laden macrophages comprise part of these hard exudates^4^,^17^. Cusick *et al*. also confirmed colocalization of lipids and macrophages in hard exudates with Oil Red O and CD68 immunostaining, respectively^6^. Our observation with AO-SLO showed that some hard exudates consisted of an accumulation of spherical particles with hyper-reflective dots, and these AO-SLO images resemble the histological images in published literature (Figs 3 and 4 in Wolter’s paper in the 1950’s)^4^. Furthermore, quantitative analysis indicated that the diameter of the spherical particles was approximately 27 μm. Generally, macrophages have been reported to be approximately 20 μm^18^. Although the spherical particles are slightly larger than a macrophage, they might represent enlarged macrophages that have phagocytosed lipid. Normal leukocytes including macrophages and monocytes are thought to be transparent to the AO-SLO light and are therefore probably not detected^19^. In contrast, it is known that lipid or proteinaceous material can be detected as hyper-reflective materials in SD-OCT. The wavelength of our AO-SLO (845 nm) is almost the same as the SD-OCT used in this study (Cirrus; 840 nm). Therefore, based on their appearance and size, the spherical particles with hyper-reflective dots in AO-SLO images could correspond to lipid-laden macrophages previously reported in pathology^4^. Furthermore, a case with hard exudate formation in DR was evaluated (Fig. 5K). The spherical particles were scattered in the baseline image of AO-SLO, however, hard exudates were invisible in the color fundus photograph. Some recent reports described the presence of hyper-reflective foci in SD-OCT with no apparent changes detected by the color fundus photograph^20^. Therefore, the spherical particles in AO-SLO might correlate with the hyper-reflective foci in SD-OCT^21^,^22^.

Various studies have shown that macrophages play an important role in various diseases^23^,^24^. In human atherosclerosis, histological insights have implicated monocyte recruitment and subsequent lipid-loading in macrophages^23^. In our study using AO-SLO, spherical particles containing many hyper-reflective cytoplasmic dots were found in retinal vascular diseases. These hyper-reflective dots are most likely to be phagocytosed lipids in macrophages. The contribution of macrophages to the pathogenesis of retinal vascular diseases might be similar to that of atherosclerosis.

Recently, the concept of DR as an inflammatory cell-related disease has gained increasing acceptance based on mounting evidence^25^,^26^. However, there is no *in vivo* evidence to support this inflammatory theory despite the fact that inflammatory cytokines are upregulated. In this study, regions with the round hard exudates (possibly representing lipid-laden macrophages) were observed to have greater macular edema. This finding suggests that inflammatory cells, in particular macrophages, might contribute not only to the pathogenesis but also to the activity of diabetic macular edema.

**Figure 7:**
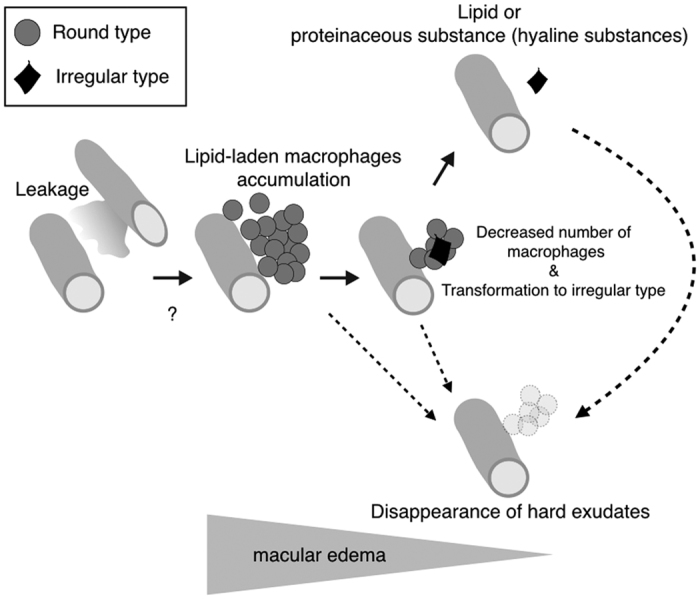
A hypothesized possible model of macrophage-related hard exudates clearance. High resolution imaging using adaptive optics scanning laser ophthalmoscopy (AO-SLO) shows two types of hard exudates. One consists of macrophages (round type) and the other could be lipid or proteinaceous materials (hyaline substances) (irregular type), respectively, as shown in Fig. 1. In retinal vascular diseases, lipid or proteinaceous materials leak from retinal vessels resulting in macular edema (Fig. 2). During the process of macular edema formation, macrophages accumulate and phagocytose leaked lipid or proteinaceous materials as shown in Fig. 5. This step could be the formation of hard exudates. Upon completion of lipid phagocytosis by macrophages, the number of macrophages then decreases (Fig. 4). If this phagocytosis is accomplished in a physiological manner, hard exudates as well as macular edema could disappear (Figs 4 and 5). If this step was dysfunctional, free lipid or protein derived from lysed phagocytes (the irregular type) could remain. However, it is unknown if irregular hard exudates can disappear without macrophages. Furthermore, it is unclear if the round hard exudates could disappear without transformation to the irregular type.

The irregular type of hard exudates as seen by AO-SLO imaging have a solid hyper-reflective pattern. Previous histological studies have also postulated that the subsequent breakdown of lipid-laden macrophages is accompanied by the disappearance of their nuclei and cell membranes that results in the presence of free lipid substances in retinal tissue^5^. These irregular hard exudates could represent free lipid or protein derived from such lysed macrophages (Fig. 7). However, it is also possible that these irregular type of hard exudates comprise leaked lipid or protein from the retinal vasculature that occurred prior to the accumulation of macrophages.

Wolter *et al*. summarized their histological findings from four eyes with hard exudates where an accumulation of fat-filled phagocytes showed different stages of degradation and the remaining cellular debris had become hyalinized in other areas^4^. In our study using AO-SLO, the same area of the retina in patients could be followed-up and it was found that the round type of hard exudates could transform to the irregular type. Therefore, our follow-up observation may be a confirmation of Wolter’s speculation from their histological findings^4^. However, in our AO-SLO study, there were some round types that disappeared without transformation to the irregular type. As the interval between our examinations was at least one month, the transformation from the round type to the irregular type might have also occurred within a month (Fig. 7). Moreover, there were a few cases whereby the irregular type of hard exudates disappeared without macrophage accumulation (Fig. 7). More frequent examinations would be necessary to reveal the detailed mechanism of hard exudate clearance.

Persistent deposition of hard exudates is often observed in patients with DR, and this can cause a decline in visual acuity^27^. However, there is currently no therapy for persistent hard exudates. Based on our observations, irregular hard exudates could account for the persistent deposition. One explanation is that the phagocytic process by macrophages may be abnormal under certain conditions (Fig. 7), as macrophages have been reported to be dysfunctional in diabetes^28^. A recent analysis from the RIDE and RISE studies showed that monthly anti-VEGF therapy for 24 months reduced the amount of hard exudates^29^. VEGF inhibition might not only affect vascular leakage, but also macrophage phagocytosis. Future therapy to activate macrophage phagocytosis might improve visual function by enhancing the removal of hard exudates. Further investigation concerning the molecular mechanisms of macrophage function in the retina would be necessary.

When focused on the underlying photoreceptor layer, we observed hard exudates to have a hyper-reflective pattern with dark areas. The dark areas were thought to be the shadows cast by the hard exudates on the cone mosaic as these dark areas disappeared when the hard exudates disappeared. Our observation of shadowing is consistent with a previous study using a Humphrey Field Analyzer where hard exudates were reported to often cause localized scotoma^30^. Some dark areas remained in the photoreceptor layer as seen by AO-SLO while some disappeared. This observation suggests that persistent hard exudates and the related retinal edema could cause damage to the retinal layer, and the light from AO-SLO might not be able to penetrate the damaged layer to reach the photoreceptor layer.

Although AO-SLO imaging is potentially useful for noninvasive assessment of hard exudates and for better understanding of the pathogenesis in retinal vascular diseases, this current study has some limitations. Our AO-SLO prototype was unable to image hard exudates in some cases with severe macular edema, vitreous opacity, and cataract. Furthermore, hard exudates were classified into only two morphological types based on their appearance. Both round and irregular types could be observed in a hard exudate in some cases. Although we classified such mixed types as round type, further investigation is needed to develop more sensitive software or an index for classification of the hard exudate type in AO-SLO imaging. Larger sample sizes are also necessary for higher-powered sensitivity and specificity determinations. Furthermore, the duration in the analysis from the follow-up observations was only four months. Longer follow-ups are necessary to delineate changes in hard exudates. Figure 7 shows a possible model concerning macrophage-related hard exudates clearance. However, this possible model is hypothetical as it is based on limited data.

In this study, we demonstrated that AO-SLO imaging detected specific *in vivo* findings related to hard exudates that were previously only seen in pathology specimens. Thus, this high resolution imaging technique may aid in further understanding of retinal diseases, and future correlations with histological and biochemical observations may lead to new therapeutic approaches.

## Methods

This study was approved by the Institutional Ethics Committees of the Kyushu University Hospital (Protocol No. 24082, UMIN000016897), and was performed in accordance with the ethical standards laid down by the Declaration of Helsinki. Written informed consent was obtained from all patients after a detailed explanation of the study.

### Participants

The study participants comprised 29 patients (32 eyes with hard exudates) who were seen at Kyushu University Hospital from May 2013 to June 2015. They included 22 patients with DR, five with retinal vein occlusion, one with hypertensive retinopathy, and one with renal retinopathy.

### Observation Procedure

At every hospital visit, patients underwent full ophthalmologic examination including best-corrected visual acuity, slit-lamp examination, dilated fundus examination, color fundus photography and spectral domain optical coherence tomography (SD-OCT) (Cirrus HD: Carl Zeiss Meditec, Dublin, CA, USA). Based on a previous report, axial length was measured in each patient using IOL master (Carl Zeiss Meditec, Jena, Germany) to calculate the AO-SLO image scale^31^. In addition, all patients underwent imaging using a prototype AO-SLO system (focal depth of approximately 60 μm) (Canon Inc., Tokyo, Japan) as described previously^32^. The AO-SLO device consists of an adaptive optics system that can measure and compensate optical aberrations produced by ocular media, a high-resolution confocal SLO imaging system, and a wide-field imaging subsystem. The wavelength of AO-SLO is 845 nm, and the wavelength of the beacon light for measurement of wavefront aberrations is 760 nm. The imaging light and the beacon light power are set at 400 and 100 μW, respectively, in accordance with the safety limits set by the American National Standards Institute. The optical resolution is 5 μm. One or two regions of hard exudates in the macular area (6 mm × 6 mm) per eye were chosen and the images were recorded for one second per scan area with a field size of 1.2° × 1.2° and 2.8° × 2.8° with the safety. The frame rate was 32 frames per second. The AO-SLO device can focus on the photoreceptor layer while compensating for aberrations of the target eye, and automatically create an en face image of the photoreceptor layer. The AO-SLO device also enables manual adjustment of the focal plane to a layer of interest where an en face image of the layer can be created. This layer adjustment range in the AO-SLO device is ±2 D from the photoreceptor layer. Therefore, the AO-SLO device allows the creation of an en face image between the photoreceptor layer and the nerve fiber layer.

### Main Outcome Measures

Forty-three discreet regions of hard exudates were analyzed using AO-SLO (500 μm × 500 μm each). In particular, images of the regions of hard exudates were focused on the retinal layer where maximum cross-section of hard exudates could be observed on the photoreceptor layer where bright cones could be clearly seen, and on the nerve fiber layer. The central fovea was defined as the location of the fixation point in the macular region. We also confirmed the localization of the central fovea from the foveal pit on SD-OCT. The classification of hard exudate morphology was performed independently by two observers (YK and YK) who were blinded to the clinical status. If a hard exudate included even one typical spherical particle, it was classified as a round type. If a hard exudate did not include any spherical particle, it was classified as an irregular type. The kappa coefficient was 0.760 (*P* < 0.001) for classification of hard exudates into the types as defined above. This result indicated good inter-observer agreement. In cases where the classifications of two observers were different, a third observer (TN), who was also masked to the clinical status, classified the hard exudate. The AO-SLO image scale was corrected using axial length by AO-SLO Retinal Image Analyzer software (ARIA; Canon Inc., Tokyo, Japan) dedicated to the prototype AO-SLO. The AO-SLO image was exported as a bitmap file and processed to an image by using Image J 1.42K (Version XX, Rasband W.S., U. S. National Institutes of Health, Bethesda, Maryland, USA, http://imagej.nih.gov/ij/, 1997–2015). Input images are converted into 8-bit grayscale image format. Intensity level of blood vessel was set to be 0 (black) manually to standardize the intensity of each image. The command path of Image > Adjust > Brightness/Contrast was used. The area in AO-SLO image where could be detected clearly by the color fundus photograph were selected by the free hand selection tool and the mean gray value was measured. The command path of Analyze > Measure was used to measure the mean gray value of the hard exudate. The area in the AO-SLO image was extracted as the hyper-reflective area which identified over the threshold of the mean gray value in each AO-SLO image. The command path of Image > Adjust > Threshold was used to differentiate the hyper-reflective area from the AO-SLO image. However, the hyper-reflective area that smaller than 30 × 30 μm^2^ was excluded in the measurement. To measure the hard exudate area, the command path of Analyze > Analyze Particles was used. We defined the area of hard exudate as the total area of hard exudates including particles surrounding the hard exudate in the AO-SLO image (500 μm × 500 μm each). This is because some hard exudate split apart while others coalesced during the follow-up period. The major axis of the spherical particles in the round type of hard exudates was measured as the diameter. To measure the diameter of the spherical particles, five particles which formed the round type of hard exudate were randomly chosen from the AO-SLO image. The straight line selection tool was used to measure the longest diameter of the particles. The command path of Analyze >Measure was used. The measurement was performed independently by two observers (MY and SN) who were blinded to the clinical status and the mean length was recorded. To measure the retinal thickness of the Early Treatment Diabetic Retinopathy Study (ETDRS) grid where the AO-SLO image was located, we used the macular cube protocol (128 horizontal line raster with 512 A-scan each, within a 6 × 6 mm^2^ area) in the Cirrus HD-OCT. Retinal thickness values (from ILM to RPE) were automatically displayed for each of the nine map sectors as defined by ETDRS.

### Follow-up study

Thirty regions in 21 eyes (19 patients: 16 DR, two RVO and one hypertensive retinopathy) could be followed up using full ophthalmologic examinations, SD-OCT and AO-SLO. The interval between observations was four months. The rate of change was calculated as a percentage based on the ratio of the area of hard exudates from the follow-up observation compared to that at the first visit.

### Statistical Analysis

Statistical analyses were performed using the software, JMP v10.0 (SAS Institute, Cary, NC). Continuous variables were analyzed using the Wilcoxon rank sum test or two-sample t-test as appropriate. Categorical variables were assessed using Fisher’s exact test. *P* values < 0.05 were considered statistically significant.

## Additional Information

**How to cite this article**: Yamaguchi, M. *et al*. High-Resolution Imaging by Adaptive Optics Scanning Laser Ophthalmoscopy Reveals Two Morphologically Distinct Types of Retinal Hard Exudates. *Sci. Rep.* **6**, 33574; doi: 10.1038/srep33574 (2016).

## Figures and Tables

**Table 1:** Clinical characteristics of patients with retinal vascular diseases.

	Age	Gender	Disease type	Eye	VA (Snellen)	HbA1c (%)	Duration of DM (year)
Case1	41	M	DR	R	20/20	7.4	3
Case2	59	M	DR	R	20/20	—	6.5
Case3	64	F	DR	L	20/125	6	15
Case4	65	M	DR	R L	20/12.5 20/20	8	6
Case5	61	F	DR	R L	20/20 20/16	6.4	12
Case6	52	F	DR	R	20/20	8.2	3
Case7	76	F	DR	L	20/200	—	17
Case8	22	F	DR	L	20/12.5	8.7	19
Case9	59	F	DR	L	20/40	7.2	6
Case10	62	M	DR	R	20/20	6	4
Case11	42	M	DR	R	20/16	10	12
Case12	65	M	DR	L	20/20	13.1	10
Case13	47	M	DR	R	20/12.5	7	12
Case14	66	F	DR	R	20/16	14	10
Case15	58	M	DR	R	20/25	7.1	7
Case16	67	F	DR	R	20/20	6.1	10
Case17	54	M	DR	R	20/63	6.5	12
Case18	70	M	DR	L	20/20	10.5	15
Case19	79	M	DR	L	20/160	—	—
Case20	76	F	DR	R	20/160	—	—
Case21	63	M	DR	R L	20/20 20/20	7.5	20
Case22	51	F	DR	R	20/32	9.3	10
Case23	84	F	BRVO	L	20/200	—	—
Case24	55	F	BRVO	L	20/20	—	—
Case25	74	F	BRVO	L	20/16	—	—
Case26	62	F	BRVO	R	20/40	—	—
Case27	75	M	BRVO	L	20/25	—	—
Case28	37	M	Hypertensive retinopathy	L	20/20	—	—
Case29	22	F	Renal retinopathy	R	20/63	—	—
