# Erratum: High-Resolution Imaging by Adaptive Optics Scanning Laser Ophthalmoscopy Reveals Two Morphologically Distinct Types of Retinal Hard Exudates

**DOI:** 10.1038/srep35127

**Published:** 2016-11-03

**Authors:** Muneo Yamaguchi, Shintaro Nakao, Yoshihiro Kaizu, Yoshiyuki Kobayashi, Takahito Nakama, Mitsuru Arima, Shigeo Yoshida, Yuji Oshima, Atsunobu Takeda, Yasuhiro Ikeda, Shizuo Mukai, Tatsuro Ishibashi, Koh-hei Sonoda

Scientific Reports 6: Article number: 3357410.1038/srep33574; published online: 09 19 2016; updated: 11 03 2016

The original version of this Article contained errors in the Abstract.

“The retinal thickness in regions with round hard exudates was significantly greater than the thickness in regions with irregular hard exudates (P = 0.01 → 0.02). This differentiation of retinal hard exudates in patients by AO-SLO may help in understanding the pathogenesis and clinical prognosis of retinal vascular diseases”.

now reads:

“The retinal thickness in regions with round hard exudates was significantly greater than the thickness in regions with irregular hard exudates (P = 0.02). This differentiation of retinal hard exudates in patients by AO-SLO may help in understanding the pathogenesis and clinical prognosis of retinal vascular diseases”.

These errors have now been corrected in the PDF and HTML versions of the Article.

